# Comparative Transcriptomics Reveals Defense Mechanisms Against *Pseudoperonospora cubensis* Infection in Cucumber Cultivars

**DOI:** 10.3390/plants15182836

**Published:** 2026-09-16

**Authors:** Qianyi Zhang, Zebin Xu, Jiran Li, Yuan Yao, Zhi Li, Tingting Yuan, Yurui Wang, Jingping Dong, Jun Xu

**Affiliations:** 1 School of Horticulture and Landscape, Yangzhou University, Yangzhou 225009, China; 2 College of Plant Protection, Yangzhou University, Yangzhou 225009, China

**Keywords:** cucumber, downy mildew, transcriptomic, differentially expressed genes, *CsJAZ8*

## Abstract

Downy mildew (DM) in cucumber (*Cucumis sativus* L.), caused by *Pseudoperonospora cubensis*, severely restricts cucumber growth and yield. This study compared transcriptomic responses to *P. cubensis* infection between the DM-resistant cucumber cultivar ‘115’ and the DM-susceptible cultivar ‘XTMC’ at 48 and 72 h post-infection. Compared with the controls, infection induced 6667 and 6601 differentially expressed genes (DEGs) in ‘115’ and ‘XTMC’ leaves, respectively. Across both time points, 97 and 90 DEGs were commonly upregulated specifically in ‘XTMC’ and ‘115’ leaves, respectively, following *P. cubensis* inoculation. Kyoto Encyclopedia of Genes and Genomes (KEGG) analysis associated genes highly expressed in each cultivar primarily with plant hormone signal transduction and plant–pathogen interaction pathways. Quantitative reverse transcription PCR (qRT-PCR) confirmed the expression patterns. *CsJAZ8* expression was downregulated in ‘115’ leaves after *P. cubensis* infection, whereas *CsJAZ8* overexpression increased cucumber susceptibility to DM. These findings provide genetic resources for investigating the mechanisms underlying DM resistance and developing molecular breeding strategies for cucumber.

## 1. Introduction

Cucumber (*Cucumis sativus* L., 2n = 2x = 14) is an economically important vegetable crop cultivated worldwide [1,2]. However, downy mildew (DM), a particularly destructive disease caused by the obligate biotrophic oomycete *Pseudoperonospora cubensis*, severely limits cucumber cultivation and production [3]. Infected cucumber leaves develop angular chlorotic lesions bounded by veins, water-soaked lesions, and purplish-black sporangia on their undersides [4]. Sporangia are dispersed mainly by wind or water and initiate repeated infection cycles. Infected leaves rapidly become necrotic, resulting in plant death and severe yield loss [5]. Cucumber DM control primarily relies on intensive fungicide use. However, excessive fungicide application can promote resistance in DM pathogen populations and substantially increase selection pressure. Extensive use of chemical fungicides can cause harmful substances to accumulate in cucumber products, adversely affecting human health and the environment [6,7]. Identifying DM resistance genes and incorporating them into breeding programs to strengthen host resistance is an efficient and sustainable strategy for controlling this disease.

Reducing the adverse effects of DM requires the continued identification of natural resistance resources and disease-resistant genes. Plants counter pathogen infection through an immune system comprising two interconnected branches: pattern-triggered immunity (PTI) and effector-triggered immunity (ETI) [8,9]. Rather than functioning as isolated pathways, PTI and ETI operate synergistically and interdependently. These overlapping responses activate mitogen-activated protein kinase pathways, pathogenesis-related (PR) gene transcription, reactive oxygen species bursts, transcriptional reprogramming, cell wall reinforcement, and the synthesis of secondary metabolites or antimicrobial compounds, including phytoalexins. Together, these processes coordinate defense responses against diverse pathogens [10,11]. The complex defense-related phytohormone network primarily comprises salicylic acid, jasmonic acid (JA), and ethylene. Hormone accumulation and signaling within this network change dynamically during PTI and ETI activation [12,13,14]. Diverse defense-related genes enable host plants to adapt to biotic stress and regulate their resistance to disease.

However, the molecular and biochemical mechanisms underlying cucumber DM resistance remain poorly understood. Several DM resistance-associated quantitative trait loci (QTLs) and candidate genes have been identified [3]. For example, cucumber *CsWRKY50* has been identified as a mediator of defense responses against DM pathogen infection [15]. In Gy14 cucumber, a single nucleotide polymorphism (SNP) in the coding region of the STAYGREEN gene (*CsSGR*) confers durable, broad-spectrum DM resistance [16]. Similarly, seven SNPs have been identified in the coding and promoter regions of cucumber *CsSIB1*, which encodes the VQ motif-containing sigma factor-binding protein 1. These SNPs occur in PI 197088 and are associated with DM resistance [17]. Although these studies provide preliminary insights into the genetic regulation of DM resistance in cucumber, the underlying molecular regulatory mechanisms remain unexplored.

Multi-omics approaches, including transcriptomics, are increasingly being used to elucidate molecular regulatory networks and host immune systems. These approaches also identify defense-related genes that can support the breeding of disease-resistant cultivars [18,19,20]. In tomato, integrated metabolomic and transcriptomic analyses have identified a pathogen-responsive biosynthetic gene cluster that facilitates the production of highly modified fatty acids and enhances plant disease resistance [21]. In cucumber, comparative physiological and transcriptomic profiling of resistant and susceptible bulks derived from an F_2_ population enabled the prediction of potential molecular mechanisms underlying DM resistance [5]. Therefore, a comparative transcriptomic analysis of resistant and susceptible cultivars is crucial for identifying defense-related genes or functional elements correlated with DM resistance.

This study identified two cucumber inbred lines with contrasting DM responses: the DM-resistant line ‘115’ and the DM-susceptible line ‘XTMC’. Their responses to *P. cubensis* infection were compared to characterize cultivar-specific differences and identify candidate DM-resistance genes. The jasmonate ZIM-domain protein gene *CsJAZ8* was downregulated in ‘115’ leaves after *P. cubensis* infection, whereas *CsJAZ8* overexpression increased cucumber susceptibility to DM. These findings advance our understanding of the molecular basis of cucumber DM resistance and support further investigation of defense-related genes to improve resistance.

## 2. Results

### 2.1. Symptoms of P. cubensis-Infected Cucumber ‘115’ and ‘XTMC’ Inbred Lines

**Figure 1:**
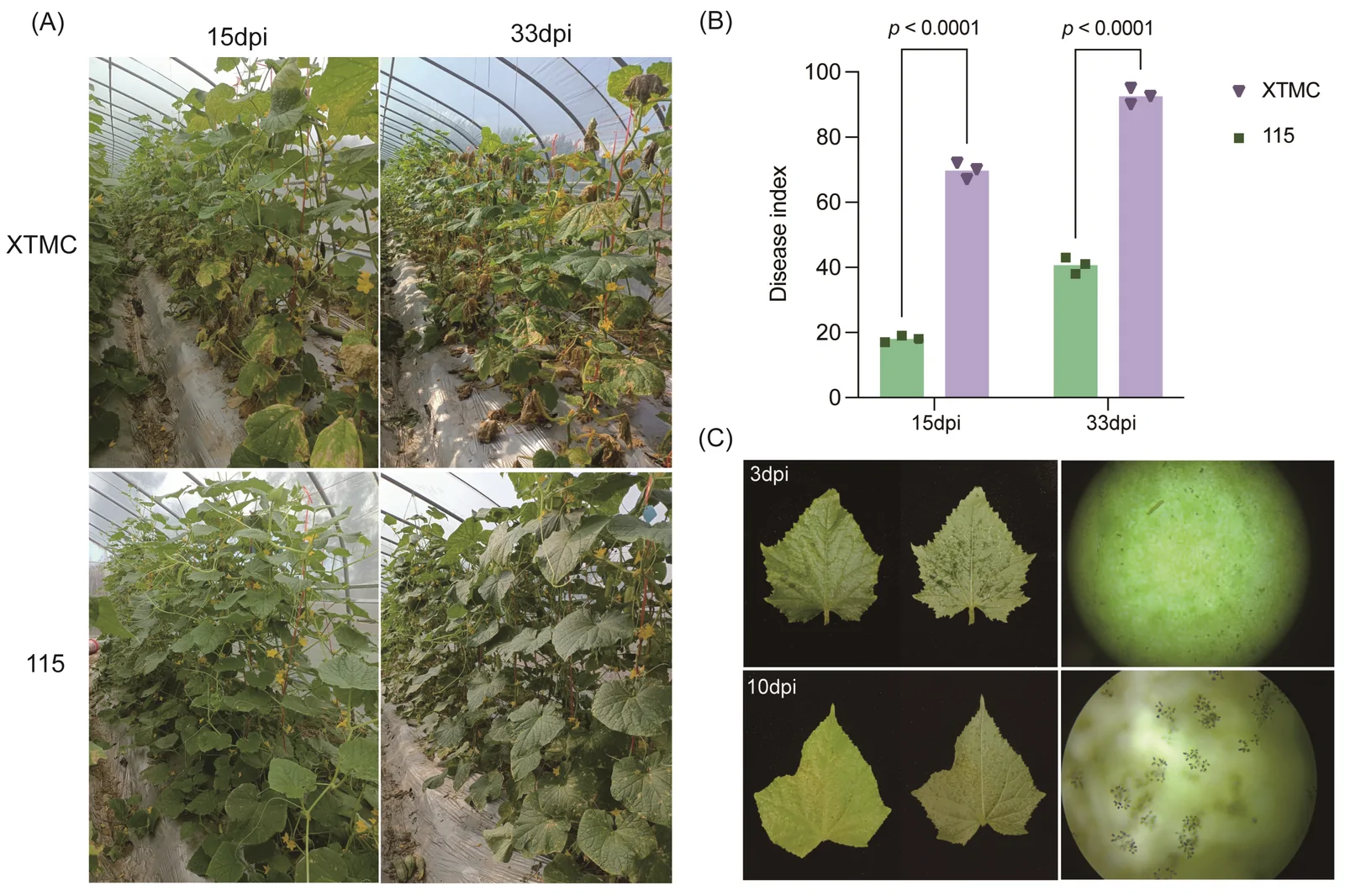
Symptoms of two cucumber cultivars infected with *P. cubensis*. (**A**) Phenotypic differences between ‘XTMC’ and ‘115’ at 15 and 33 days after *P. cubensis* inoculation. (**B**) Disease indices of ‘XTMC’ and ‘115’ at 15 and 33 days after *P. cubensis* inoculation. (**C**) Differences in symptoms between ‘XTMC’ and ‘115’ seedlings at 3 and 10 days post-inoculation. The vertical axis represents the mean disease index, and the horizontal axis represents the days post-inoculation. Data are presented as the mean ± standard deviation of three biological replicates, with at least 20 seedlings evaluated per replicate. Significant differences were assessed using Dunnett’s multiple comparison test (‘XTMC’ inoculation vs. ‘115’ inoculation, *n* = 3).

The progression of DM symptoms following *P. cubensis* inoculation was assessed in the cucumber inbred lines ‘115’ and ‘XTMC’ at the seedling and maturity stages. In mature plants, ‘XTMC’ developed larger yellow angular lesions or displayed leaf wilting at 15 and 33 days post-inoculation (dpi). In contrast, ‘115’ showed only mild symptoms with few yellow angular lesions at 33 dpi (Figure 1A). At both inoculation stages, ‘115’ consistently exhibited a markedly lower disease severity index than ‘XTMC’ (Figure 1B). During the seedling assay, a small number of water-soaked lesions accompanied by spores appeared on ‘XTMC’ leaves at 3 dpi. By 10 dpi, these initial lesions had expanded into yellow-brown angular lesions with gray-black growth comprising spores and mycelium (Figure 1C). Thus, 3 dpi may be a critical time point for investigating resistance genes involved in effective defense against DM. The contrasting symptom profiles confirmed a substantial difference in DM resistance between ‘115’ and ‘XTMC’.

### 2.2. Transcriptome Analysis of Cucumber ‘115’ and ‘XTMC’ Inbred Lines Under P. cubensis Stress

**Figure 2:**
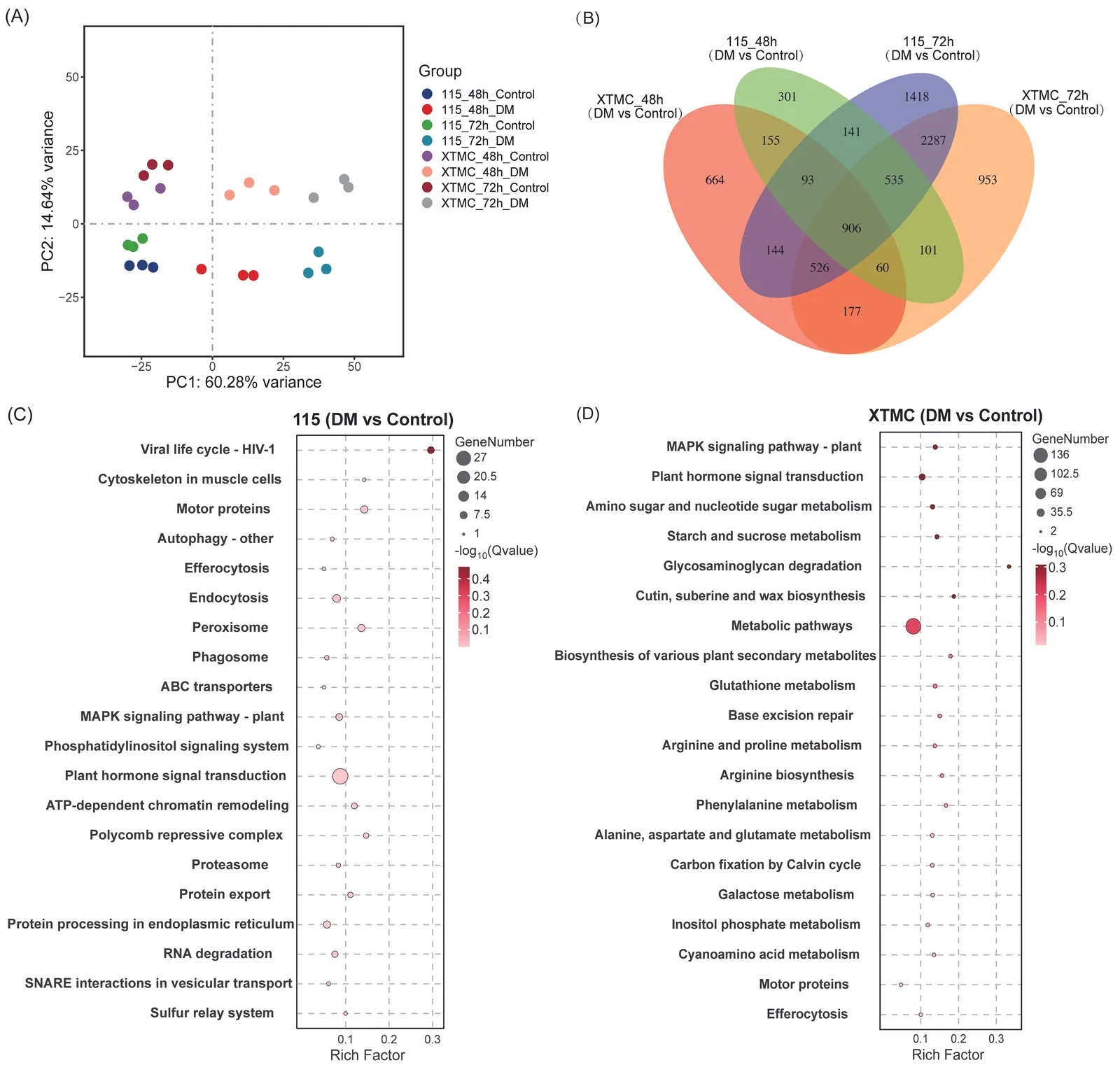
Transcriptome analysis of ‘115’ and ‘XTMC’ following *P. cubensis* infection. (**A**) Principal component analysis (PCA) of samples from both cultivars. (**B**) Venn diagram analysis of differentially expressed genes (DEGs) in both cucumber lines inoculated with *P. cubensis*. (**C**,**D**) Kyoto Encyclopedia of Genes and Genomes (KEGG) enrichment analysis of DEGs specifically detected in the cucumber lines ‘115’ and ‘XTMC’, respectively. Functional classification was performed on the annotated DEGs.

Comparative transcriptome profiling of leaves collected at 48 and 72 h after *P. cubensis* inoculation was conducted to examine the molecular basis of DM resistance in ‘115’ and ‘XTMC’. In the control (ddH_2_O-treated) and inoculated (DM) groups, 24 cDNA libraries generated more than 65 GB of clean sequencing data. Over 94% of bases had Phred quality scores ≥ 30, and the mean guanine–cytosine content was 43% (Supplementary Table S2). Biological replicates clustered closely in the principal component analysis (PCA), whereas inoculation status and cultivar identity clearly separated the samples (Figure 2A). DEGs were identified using |log_2_ (fold change)| ≥ 1 and FDR < 0.05 by comparing infected leaves with their corresponding uninfected controls at both sampling times (Supplementary Figure S1). Venn diagram analysis of the four comparison groups revealed substantial overlap among transcriptional responses across time points and cultivars. Overall, *P. cubensis* infection induced 6667 DEGs in ‘115’ and 6601 DEGs in ‘XTMC’ compared with their respective controls (DM vs. control) (Figure 2B). KEGG analysis showed that these DEG sets were predominantly enriched in metabolic pathways and secondary metabolite biosynthesis (Supplementary Figure S2). Cultivar-specific responses included 1860 DEGs in ‘115’ and 1794 DEGs in ‘XTMC’. KEGG enrichment further distinguished these cultivar-specific DEG sets. Genes unique to ‘115’ were primarily associated with plant hormone signal transduction, whereas those specific to ‘XTMC’ were mainly associated with metabolic pathways (Figure 2C,D).

### 2.3. Identification of DEGs Between ‘115’ and ‘XTMC’ After P. cubensis Inoculation

**Figure 3:**
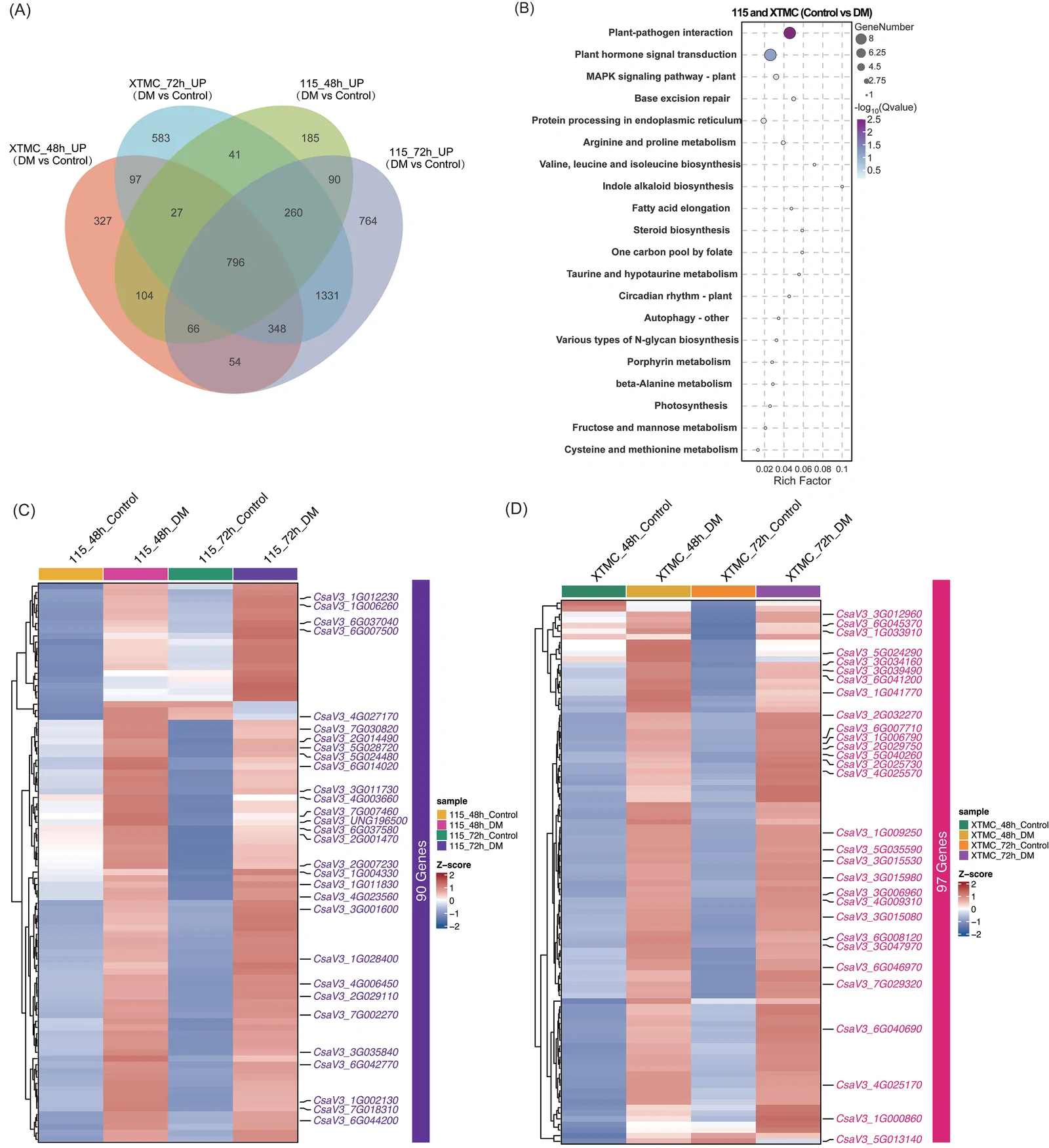
Analysis of DEGs in cucumber ‘115’ and ‘XTMC’ after *P. cubensis* inoculation. (**A**) Venn diagram analysis of upregulated DEGs in the two inoculated cucumber cultivars. (**B**) KEGG analysis of 97 and 90 DEGs commonly upregulated at both time points specifically in ‘XTMC’ and ‘115’ leaves, respectively, following *P. cubensis* inoculation. Functional annotation was performed on all 187 DEGs. (**C**,**D**) Expression profiles of 90 and 97 upregulated DEGs specific to cucumber ‘115’ and ‘XTMC’, respectively, following *P. cubensis* inoculation. The color scale represents differences in gene transcript levels.

To investigate transcriptional variation underlying defense responses to *P. cubensis* infection, responses were compared across time points and between cucumber cultivars ‘115’ and ‘XTMC’. Compared with non-inoculated controls, ‘XTMC’ contained 1819 and 3483 upregulated DEGs at 48 and 72 h post-inoculation, respectively. At the corresponding time points, ‘115’ contained 1569 and 3709 upregulated DEGs (Figure 3A). At both time points, 97 and 90 DEGs were commonly upregulated specifically in ‘XTMC’ and ‘115’ leaves, respectively, following *P. cubensis* inoculation (Figure 3A). KEGG enrichment analysis indicated that the upregulated genes shared by both cultivars were mainly enriched in plant–pathogen interaction and plant hormone signal transduction pathways (Figure 3B). Heatmap visualization and hierarchical clustering separately displayed 97 and 90 upregulated DEGs with significant expression changes in the ‘XTMC’ and ‘115’ leaves, respectively (Figure 3C,D). These findings revealed substantial changes in DEGs associated with plant–pathogen interactions and plant hormone signal transduction in both cultivars following *P. cubensis* inoculation.

### 2.4. Validation of Expression Patterns of Candidate DEGs Using qRT-PCR

**Figure 4:**
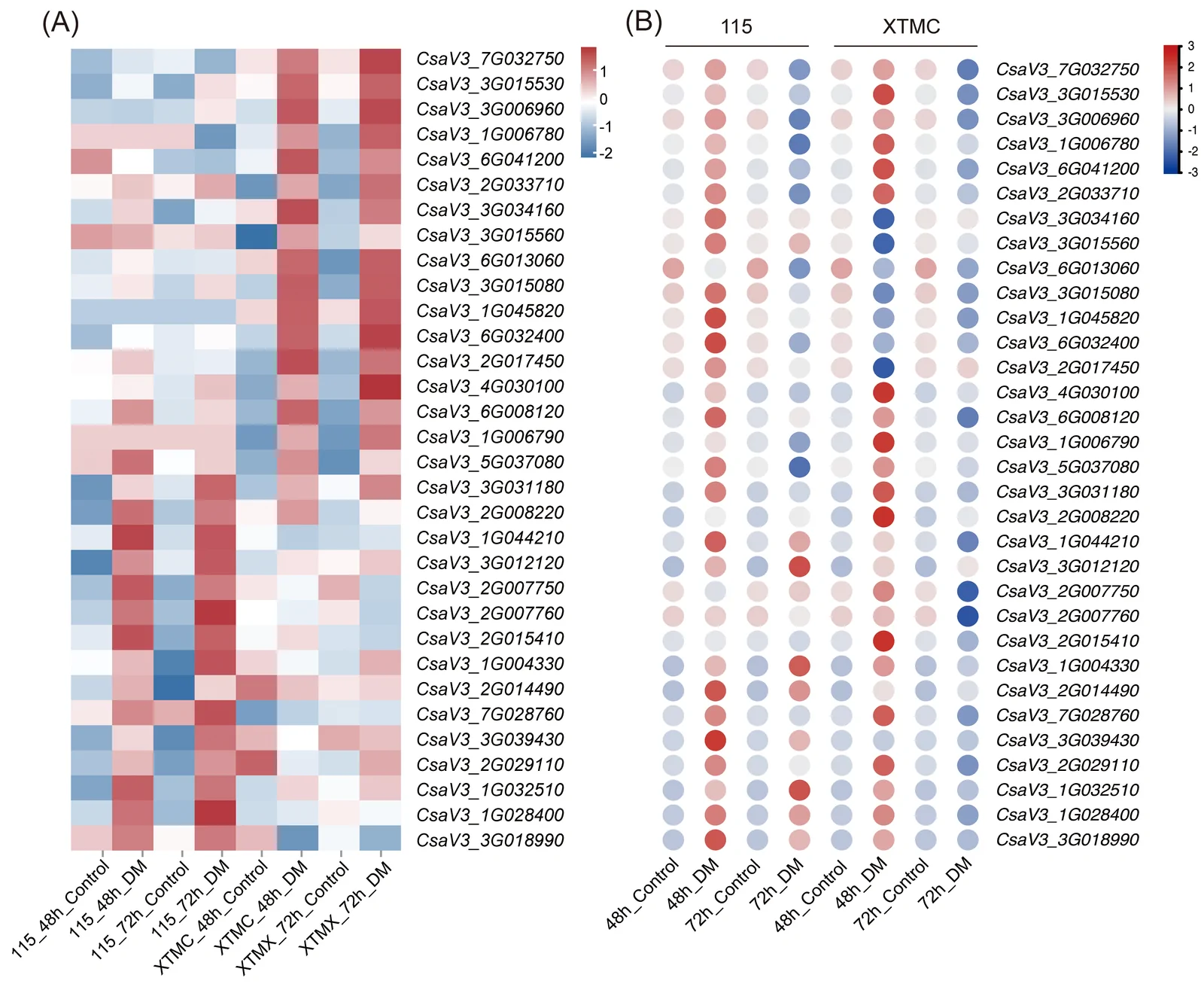
Expression of candidate genes in response to *P. cubensis*. (**A**) Expression profiles of 32 candidate DEGs specific to cucumber ‘115’ and ‘XTMC’, following *P. cubensis* inoculation. (**B**) Candidate gene transcript abundance was calculated using the 2^−ΔΔCt^ method and normalized for heatmap generation. The color scale represents differences in gene expression.

The expression patterns of 32 candidate DEGs were further examined using qRT-PCR in *P. cubensis*-inoculated leaves of DM-resistant line ‘115’ and the DM-susceptible line ‘XTMC’. qRT-PCR detected significant differential expression of these candidate genes, and demonstrated good concordance between the expression trends of these candidate genes from qRT-PCR and RNA-seq FPKM values in the resistant line ‘115’, with optimal consistency observed at 72 h post-treatment. In the susceptible line ‘XTMC’, the expression trends from transcriptomic data and qRT-PCR results were basically consistent for most genes; however, several genes showed opposing expression trends specifically at the 72 h post-treatment time point (Figure 4A,B and Supplementary Table S3). These qRT-PCR results demonstrate distinct expression responses between the cultivars among genes involved in defense against *P. cubensis* (Figure 4B). Seven genes in DM-susceptible line ‘XTMC’ showed pronounced upregulation after inoculation. These genes were barely induced or even downregulated in the resistant line ‘115’ at the same inoculation timepoints, indicating that their activation is associated with susceptibility to DM. Among them, four genes encoded a DNA-binding protein (CsaV3_3G015530), an alpha/beta hydrolase (CsaV3_6G041200), a serine/threonine-protein kinase (CsaV3_4G030100), and a disease-resistance locus receptor-like protein kinase (CsaV3_1G006790). The remaining genes encoded jasmonate ZIM-domain protein 8 (CsaV3_5G037080), zinc-finger protein CONSTANS-LIKE 5 (CsaV3_2G008220), and auxin-responsive protein SAUR23 (CsaV3_2G015410), respectively. Notably, alpha/beta hydrolase and zinc-finger protein CONSTANS-LIKE 5 were upregulated more than 20-fold in XTMC_48h_DM compared with the control (Figure 4B and Supplementary Table S3). A different expression profile was observed in DM-resistant line ‘115’, with 11 genes showing both significant upregulation and high expression following *P. cubensis* inoculation. These genes were not strongly activated in ‘XTMC’, suggesting that they may contribute to the enhanced defense response of the resistant line upon pathogen attack. Five genes included a phosphate transporter (CsaV3_3G034160), which warrants particular attention, diacylglycerol acyltransferase (CsaV3_3G015560), chitinase-like protein (CsaV3_3G015080), zinc-binding dehydrogenase family protein (CsaV3_6G032400), and calcium uniporter protein 2 (CsaV3_2G017450). The remaining genes encoded proton pump-interactor 1 (CsaV3_6G008120), phosphoenolpyruvate carboxylase kinase 4 (CsaV3_3G012120), phosphoethanolamine N-methyltransferase (CsaV3_1G004330), non-symbiotic hemoglobin (CsaV3_2G014490), a pleiotropic drug resistance ATP-binding cassette (ABC) transporter (CsaV3_3G039430), and chlorophyll a/b binding protein (CsaV3_1G032510). Collectively, these contrasting transcriptional responses between the two cucumber cultivars uncover sets of candidate susceptibility and resistance genes that respond differentially to *P. cubensi* infection.

### 2.5. Overexpression of CsJAZ8 Increases Susceptibility to DM

**Figure 5:**
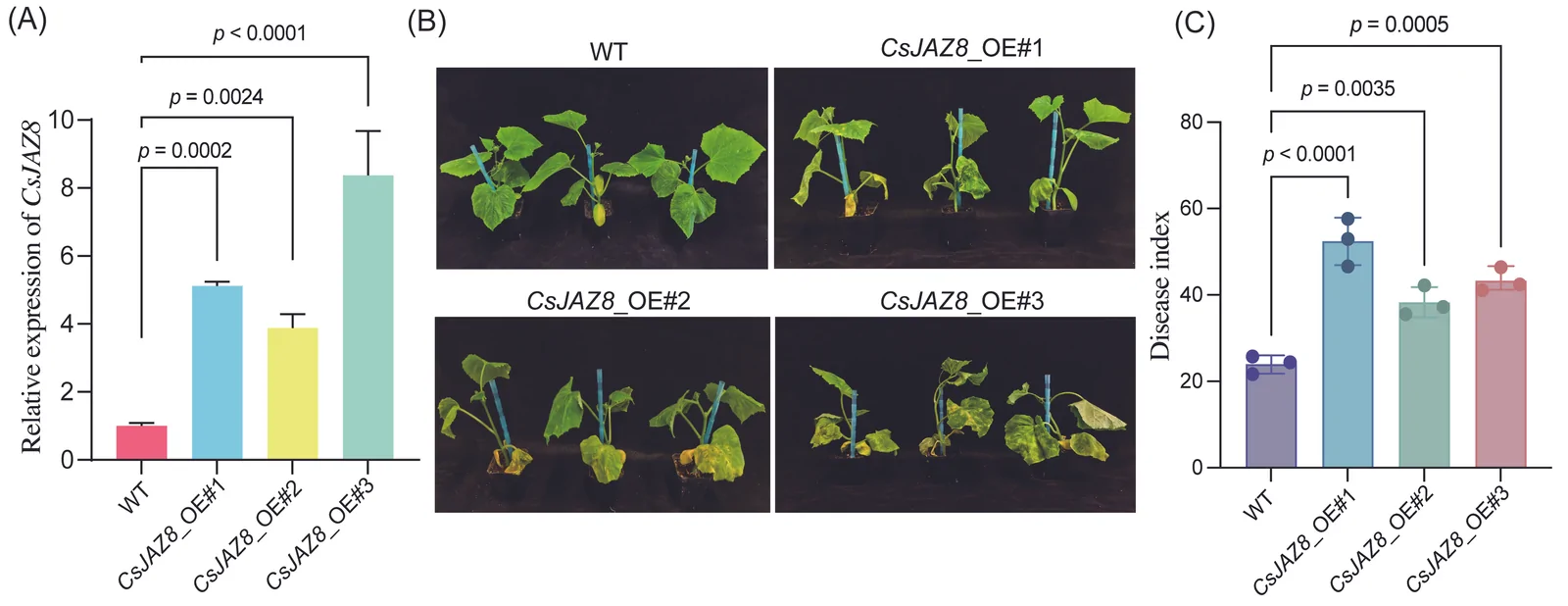
Overexpression of *CsJAZ8* increases cucumber susceptibility to downy mildew. (**A**) *CsJAZ8* expression in overexpression (OE) lines (OE1, OE2, and OE3) and wild type (WT, 9930). (**B**) Phenotypes of WT and *CsJAZ8*-overexpressing cucumber plants at 10 days post-inoculation with *P. cubensis*. Mock control plants were treated with double-distilled water. (**C**) Disease indices of WT and *CsJAZ8*-overexpressing plants following *P. cubensis* inoculation. The vertical axis represents the mean disease index, and the horizontal axis indicates the OE lines and WT. Values are presented as mean ± standard deviation of three biological replicates. Significant differences were analyzed using Dunnett’s multiple comparison test (OE lines vs. WT, *n* = 3).

Our previous research showed that *CsJAZ8* overexpression negatively regulated adventitious root formation in cucumber under waterlogging stress [22]. To functionally characterize the candidate genes identified above, subsequent investigation focused on *CsJAZ8* and its contribution to cucumber resistance against DM. Initial analysis of three overexpression lines (OE1, OE2, and OE3) and the wild type (WT, 9930) showed significantly higher *CsJAZ8* expression in the *CsJAZ8*_OE lines than in WT plants (Figure 5A). *CsJAZ8*-overexpressing and WT seedlings were sprayed with *P. cubensis* at a final concentration of 1 × 10^5^ spores/mL. At 10 days after inoculation, *CsJAZ8*-overexpressing seedlings developed angular chlorotic lesions bounded by leaf veins. Compared with WT plants, these seedlings developed conspicuous water-soaked lesions and purplish-black sporangia on the underside of their leaves (Figure 5B). Consistent with these phenotypes, *CsJAZ8*-overexpressing seedlings had a markedly higher disease index than WT plants after *P. cubensis* inoculation (Figure 5C). These findings demonstrated that *CsJAZ8* overexpression increased cucumber susceptibility to DM, implying that the candidate genes were induced to mediate DM resistance in ‘XTMC’ and ‘115’.

## 3. Discussion

Among cucumber foliar diseases, DM, caused by the oomycete *Pseudoperonospora cubensis* (Berk. & Curt.) Rostov., is one of the most destructive. This pathogen infects a broad range of cucurbit hosts worldwide and causes substantial economic loss [23,24]. Consequently, the control of DM has received increasing attention. During *Phytophthora* infection, apoplastic and cytoplasmic effectors are secreted and target distinct subcellular compartments and signaling pathways within host plant cells [25]. Several Arg-X-Leu-Arg (RxLR) effectors have been recently shown to modulate plant defense responses during *Phytophthora capsici* infection [26]. RxLR effector genes have also been identified in the *P. cubensis* genome. These effectors were cloned and functionally characterized using transient expression assays in *Nicotiana benthamiana* leaves. These assays demonstrated that several effectors suppressed programmed cell death, thereby contributing to pathogen-mediated suppression of host immunity [27]. These effectors have primarily been used to identify cucumber resistance resources, whereas breeding applications remain in the fundamental research stage.

Microbial approaches to DM control have also been investigated, and potential antagonistic microorganisms have been identified and applied [28]. For example, *Bacillus velezensis* YL2021 produces siderophores and has shown potential as a biocontrol agent for DM management [29]. Chemical control remains common, and numerous fungicides are used against cucumber DM [7]. The pyrimidinamine compound 1c has a low half-maximal effective concentration (EC_50_) of 0.10 mg/L and high fungicidal activity against DM [6]. Oxathiapiprolin was registered in China in 2015 to manage *Phytophthora infestans*, *Phytophthora capsici*, *Pseudoperonospora cubensis*, and *Plasmopara viticola* [30]. The novel pyrazole-5-carboxamide derivative T24 (EC_50_ = 0.88 mg L^−1^) is a potential fungicide for controlling *P. cubensis* [31]. However, the risk of *P. cubensis* developing resistance to different fungicides and the systemic translocation of these compounds within host plants remain poorly understood. Previous genetic studies have identified several QTLs and candidate genes that confer or contribute to DM resistance in cucumber. Dissection of the DM-resistance QTL *DM4.1* on cucumber chromosome 4 revealed multiple sub-QTLs. Evidence from fine mapping and heterologous expression analyses further supports *CsLRK10L2* as the causal gene underlying the *P. cubensis* resistance conferred by sub-QTL *DM4.1.2* [32]. Despite the substantial expansion of DM control methods, pathogens have become more aggressive, fungicide-resistant, and heat-tolerant [33,34,35,36].

Using resistant cultivars to identify disease-resistant genes and develop DM-resistant varieties represents the most sustainable approach for DM control. In this study, the DM-resistant cucumber line ‘115’ and the DM-susceptible line ‘XTMC’ were identified based on their responses to *P. cubensis* infection (Figure 1). Comparing transcriptomic responses between resistant and susceptible cultivars identified expression patterns associated with resistance or susceptibility, indicating important functions for these genes in host defense [37,38]. At 48 and 72 h after *P. cubensis* infection, 97 and 90 DEGs were significantly induced and upregulated in ‘XTMC’ and ‘115’ leaves, respectively. KEGG enrichment analysis indicated that highly expressed DEGs were significantly associated with plant hormone signal transduction and plant–pathogen interaction pathways (Figure 3). Among genes associated with fungal defense, two genes encoding alpha/beta hydrolase and zinc finger protein CONSTANS-LIKE 5 were upregulated by more than 20-fold in XTMC_48h_DM compared with the control. The jasmonate ZIM-domain protein *CsJAZ8* also showed markedly lower expression in the 115_72h_DM samples (Figure 4). Jasmonate ZIM-domain (JAZ) proteins are key regulators of the JA signaling pathway and have central roles in plant responses to biotic stress. Overexpression of *IbJAZ10* in sweet potato increases susceptibility to soft rot [39]. Similarly, cucumber plants transiently overexpressing *CsJAZ2*, *CsJAZ6*, or *CsZML2* show increased susceptibility to *Botrytis cinerea* infection [40]. In the present study, *CsJAZ8* overexpression increased cucumber susceptibility to DM (Figure 5), supporting the role of *CsJAZ8* as a negative modulator of the cucumber–*P. cubensis* interaction. This finding is consistent with previous reports that the overexpression of certain JAZ homologs increases plant susceptibility to oomycete pathogens. *CsJAZ8* may interfere with JA-triggered immune signaling, thereby suppressing defense responses, although its direct interaction partners remain to be characterized.

Our transcriptomic data also identified several additional defense-associated genes whose expression changed in the DM-resistant line ‘115’ during *P. cubensis* infection. Three genes were upregulated by more than 10-fold in 115_48h_DM or 115_72h_DM compared with the controls, implying that they positively regulated plant defense responses (Figure 4 and Supplementary Table S3). These genes encoded non-symbiotic hemoglobin (CsaV3_2G014490), a pleiotropic drug-resistant ABC transporter (CsaV3_3G039430), and chlorophyll a/b-binding protein (CsaV3_1G032510). Previous studies have shown that ectopic overexpression of the cotton non-symbiotic hemoglobin *GhHb1* in *Arabidopsis* upregulates the defense-related genes *PR-1* and *PDF1.2*. This overexpression also increased resistance to *Pseudomonas syringae* and tolerance to *Verticillium* wilt [41]. OsPDR1 belongs to the pleiotropic drug resistance subfamily of rice ABC transporters. It can modulate JA biosynthesis, activate defense-related genes, and enhance plant growth and resistance to bacterial blight [42]. Tomato chlorophyll a/b-binding protein gene *SlLhcb13* exhibits post-transcriptional upregulation despite transcriptional repression, and overexpression of *SlLhcb13* enhances resistance to *B. cinerea*, indicating that *SlLhcb13* acts as a positive regulator linking photosynthesis and immunity [43]. Collectively, our results reveal divergent gene expression profiles in DM-susceptible versus DM-resistant cucumber lines following *P. cubensis* infection and provide preliminary evidence for the roles of these candidates in plant defense. Although these candidate genes require functional validation, transcriptome-derived candidates provide a valuable resource for dissecting cucumber–*P. cubensis* interactions and supporting future marker-assisted breeding. Future studies will generate transgenic cucumber lines by knockdown and overexpression of these candidate genes. These lines will help elucidate the underlying molecular mechanisms and facilitate the breeding of DM-resistant cucumber cultivars.

## 4. Materials and Methods

### 4.1. Plant Materials and Treatment

The cucumber materials comprised the DM-resistant inbred line ‘115’, the DM-susceptible inbred line ‘XTMC’, three *CsJAZ8*-overexpressing (OE) lines, and the wild-type 9930. These materials were bred in the laboratory and cultivated in the experimental field at Yangzhou University, Yangzhou, China. Cucumber seedlings were subsequently grown in a controlled chamber under a 16 h light/8 h dark photoperiod, with day and night temperatures of 25 °C and 18 °C, respectively. Naturally infected cucumber leaves collected from the experimental field were used to obtain and isolate the *P. cubensis* strain. For seedling inoculation, the resulting spore suspension was adjusted to 1 × 10^5^ spores/mL using sterile distilled water and applied at the second true-leaf stage. Mock controls received sterile double-distilled water (ddH_2_O) instead of the spore suspension and were maintained separately under otherwise identical conditions. Successful inoculation was verified through continuous microscopic and phenotypic monitoring of infected leaves combined with trypan blue staining. Leaves from the DM and mock control groups were collected at 48 and 72 h after infection, with three biological replicates at each time point. Samples were designated as 115_48h_DM, 115_72h_DM, 115_48h_control, 115_72h_control, XTMC_48h_DM, XTMC_72h_DM, XTMC_48h_control, and XTMC_72h_control, respectively. For disease index assessment, the cucumber inbred line ‘9930’ and three *CsJAZ8*-overexpressing lines (OE1, OE2, and OE3) were spray-inoculated with *P. cubensis* at 1 × 10^5^ spores mL^−1^. Each experimental group included at least 20 individual seedlings per biological replicate, with three independent biological replicates. Disease severity was scored using a modified grading scale based on the percentage of symptomatic leaf area [4].

### 4.2. RNA Extraction and Transcriptome Sequencing

Total RNA was isolated using TRIzol reagent (Invitrogen, Carlsbad, CA, USA) according to the manufacturer’s protocol. RNA purity, concentration, and integrity were evaluated using an Agilent 2100 Bioanalyzer (Agilent Technologies, Santa Clara, CA, USA) and a NanoDrop 2000 spectrophotometer (Thermo Scientific, Waltham, MA, USA). High-quality RNA was sequenced on the Illumina NovaSeq 6000 platform by Oebiotech Company (Shanghai OE Biotech Co., Ltd., Shanghai, China).

### 4.3. Gene Expression Quantification and Differential Analysis

Raw reads in FASTQ format were processed using fastp, and low-quality reads were removed to generate clean reads [44]. HISAT2 was then used to map the clean reads to the cucumber reference genome obtained from the Cucurbit Genomics Database (http://www.cucurbitgenomics.org/v2/, accessed on 15 August 2026) [45]. Gene expression was quantified as fragments per kilobase of transcript per million mapped reads (FPKM), whereas HTSeq-count generated raw read counts [46,47]. Similarity among biological replicates was assessed using principal component analysis (PCA) in R version 3.2.0.

Differentially expressed genes (DEGs) were identified using DESeq2 [48]. The Benjamini–Hochberg method was used to adjust the *p*-values to control the false discovery rate (FDR). Genes with an FDR < 0.05 and |log_2_(fold change)| ≥ 1 were classified as DEGs [18]. Kyoto Encyclopedia of Genes and Genomes (KEGG) enrichment analysis was performed to identify significantly enriched terms among the DEGs. R version 3.2.0 was used to present the enrichment results as bar charts, chord diagrams, and bubble plots [49,50]. FPKM values for candidate DEGs were analyzed by heatmap visualization and hierarchical clustering using TBtools -II (v2.390) [51].

### 4.4. Quantitative Reverse Transcription PCR (qRT-PCR) Analysis

Total RNA was reverse-transcribed using HiScript Q RT SuperMix for qPCR (+gDNA wiper) (Vazyme, Nanjing, China) to generate complementary DNA (cDNA) for qRT-PCR. Gene-specific primers targeting candidate cucumber genes and the tubulin reference gene (CsaV3_4G000060) were designed using Beacon Designer 8.14. The sequences are listed in Supplementary Table S1. Amplification was performed using an iQ^TM^5 Multicolor qPCR Detection System (Bio-Rad, Hercules, CA, USA). Each 20 μL reaction contained AceQ SYBR Green Master (Low ROX Premixed) (Vazyme, Nanjing, China). Each biological sample was analyzed in three technical replicates. Relative transcript levels of the candidate genes were calculated using the 2^−ΔΔCt^ method, with tubulin serving as the internal reference.

### 4.5. Data Analysis

Statistical analyses were performed using SPSS version 20.0. Differences between control and treatment groups were evaluated using Dunnett’s multiple-comparison test. Comparative cucumber leaf transcriptome data were deposited in the National Center for Biotechnology Information (NCBI) database under the accession number PRJNA1512099.

## 5. Conclusions

Comparative analysis of transcriptomic profiles and qRT-PCR data from the DM-resistant cucumber line ‘115’ and the DM-susceptible line ‘XTMC’ identified DM-responsive genes after *P. cubensis* inoculation. KEGG enrichment analysis identified and annotated the functions of candidate genes contributing to *P. cubensis* resistance. *CsJAZ8* was selected from these candidates because its expression was downregulated in ‘115’. Transgenic validation demonstrated that *CsJAZ8* negatively regulated cucumber resistance to DM. These findings provide genetic resources for investigating the molecular mechanisms underlying cucumber DM resistance and breeding DM-resistant cultivars.

## Data Availability

The transcriptome data was deposited in NCBI under accession PRJNA1512099, and other data supporting the findings of this study are available in the references cited in this manuscript.

## Supplementary Materials

The following supporting information can be downloaded at: https://www.mdpi.com/article/10.3390/plants15182836/s1, Figure S1. Differentially expressed gene (DEG) counts in cucumber ‘115’ and ‘XTMC’ following *P. cubensis* inoculation or mock treatment with double-distilled water; Figure S2. Kyoto Encyclopedia of Genes and Genomes (KEGG) analysis of DEGs in ‘115’ and ‘XTMC’ leaves after *P. cubensis* inoculation; Table S1. Information of the PCR primers; Table S2. The transcriptome clean data summary; Table S3. Quantitative reverse transcription PCR (qRT-PCR) and FPKM values for candidate DEGs.

## Abbreviations

ABC: ATP-binding cassette; cDNA: complementary DNA; ddH_2_O: double-distilled water; DEG: differentially expressed gene; DM: downy mildew; ETI: effector-triggered immunity; FDR: false discovery rate; FPKM: fragments per kilobase of transcript per million mapped reads. JA: jasmonic acid; JAZ: jasmonate ZIM-domain; KEGG: Kyoto Encyclopedia of Genes and Genomes; NCBI: National Center for Biotechnology Information; OE: overexpression; PCA: principal component analysis; PCR: polymerase chain reaction. PDR: pleiotropic drug resistance; PMMoV: pepper mild mottle virus; PR: pathogenesis-related; PTI: pattern-triggered immunity; qPCR: quantitative polymerase chain reaction; qRT-PCR: quantitative reverse transcription PCR; QTL: quantitative trait locus; RxLR: Arg-X-Leu-Arg; SNP: single-nucleotide polymorphism; WT: wild type.
